# Hepaticocholecystoduodenostomy compared with Roux-en-y choledochojejunostomy for decompression of the biliary tract

**DOI:** 10.4103/0256-4947.55169

**Published:** 2009

**Authors:** Omar Shah, Parveen Shah, Showkat Zargar

**Affiliations:** a From the Department of Surgery, Sher-i-Kashmir Institute of Medical Science, Srinagar, Kashmir, India; b From the Department of Pathology, Sher-i-Kashmir Institute of Medical Science, Srinagar, Kashmir, India; c From the Department of Gastroenterology, Sher-i-Kashmir Institute of Medical Science, Srinagar, Kashmir, India

## Abstract

**BACKGROUND AND OBJECTIVES:**

The nature of palliative decompressive surgery for unresectable periampullary tumor is usually determined by the experience of the surgeon. We compared hepaticocholecystoduodenostomy (HCD), a new palliative decompressive anastomotic technique, to Roux-en-y choledochojejunostomy (CDJ) in this prospective, randomized study.

**PATIENTS AND METHODS:**

Twenty patients who were to undergo surgery for palliative bypass were randomized into two groups: group I was subjected to HCD (10 patients) and group II to CDJ (10 patients). Pre- and postoperative liver function tests, operative time, operative blood loss, onset of postoperative enteral feeding, length of hospital stay and survival rates were compared in the two groups.

**RESULTS:**

Effective surgical decompression was observed clinically as well as on analysis of pre- and postoperative liver function tests in both the groups. The results were statistically significant in favor of patients in group I when compared to those of group II with respect to operative time 84.7 (10.3) min vs 133.6 (8.9) min; *P* =<.0001), operative blood loss 137.8 (37.2) mL vs 201.6 (23.4) mL; *P* =.001), postoperative enteral feeding 3.3 (0.5) days vs 5.0 (0.7) days; *P*=<.0001) and length of hospital stay 7.5 (0.7) days vs 9.7 (1.2) days; *P*=<.0001). During follow-up, recurrent jaundice was observed in one patient in group I and two patients in group II, while duodenal obstruction developed in one patient in the group I series. Gastrointestinal hemorrhage occurred in one patient belonging to group II. The difference in mean survival time was not statistically significant.

**CONCLUSION:**

Based on this small series, HCD seems to be a better palliative surgical procedure than the routinely performed CDJ.

Periampullary cancer may arise from the head of the pancreas, the ampulla of Vater, the lower part of the common bile duct or the duodenal mucosa. Often due to the late presentation of the disease, patients are subjected to palliative therapy with a view to relieve obstructive jaundice, gastric outlet obstruction and pain.^1^–^3^ Operative and nonoperative modalities are currently available to provide reasonable palliation of symptoms. Appropriate palliative surgical management is indicated in patients found to have unresectable tumors at the time of laparotomy or those whose symptoms are not amenable to current nonoperative palliative techniques. Various surgical bypass procedures are performed for relief from obstructive jaundice. The most frequently used procedures are hepaticojejunostomy, cholecystojejunostomy, choledochojejunostomy and choledochoduodenostomy. To our knowledge hepaticocholecystoduodenostomy (HCD), a palliative decompression procedure has not been attempted in such cases. This study attempts to introduce the procedure of HCD as a decompressive procedure in such cases. HCD involves the interposition of the gallbladder between the common hepatic duct and the second part of duodenum to facilitate the free drainage of bile. In this study, we compared the outcome of this procedure (HCD) with the routinely performed choledochojejunostomy (CDJ) to assess the HCD as a new viable procedure for palliative decompression in unresectable cases of periampullary tumors.

## PATIENTS AND METHODS

The patients included in this study had unresectable and histologically proven periampullary adenocarcinoma detected preoperatively (included patients with advanced disease where biliary stenting has failed or stents were repeatedly blocked) or intraoperatively by finding of fixed tumors non-amenable for palliative surgical bilioenteric bypass. These patients were enrolled in the Department of Surgical Gastroenterology at Sher-i-Kashmir Institute of Medical Sciences, Srinagar, Kashmir, India, for this study, which extended from January 2004 to January 2008. The diagnosis of the tumor was based on clinical examination and was aided by ultrasonography (US), CT and endoscopic retrograde cholangiopancreatography (ERCP). Radiologically the criteria for determining resectability included the presence or absence of distant metastases and local invasion of the major retroperitoneal vascular structures, especially the portal and superior mesenteric veins and the superior mesenteric artery. Histological proof was obtained either by tissue biopsy on lateral duodenoscopy or by USG/CT-guided fine needle aspiration biopsy (FNA) of the tumor mass. Patients with a history of endoscopic biliary drainage, prior biliary surgery or gall stone disease were excluded from the study.

Randomization of patients was carried out intraoperatively based on random number allocation after the surgeon declared the tumor as unresectable. Patients were assigned HCD (group I), or CDJ (group II) by a technologist in the operating theatre after opening an opaque sealed envelope. Inclusion criteria were a dilated common bile duct, distended gallbladder, no tumor encroachment of the terminal cystic duct, no features of duodenal obstruction, and sufficient grounds to believe that either procedure would be technically safe. For comparison, relevant details including pre- and postoperative liver function test results, operative time, operative blood loss, onset of enteral feeding, length of hospital stay and survival rates were recorded in each group.

### Surgical technique

**Figure 1:**
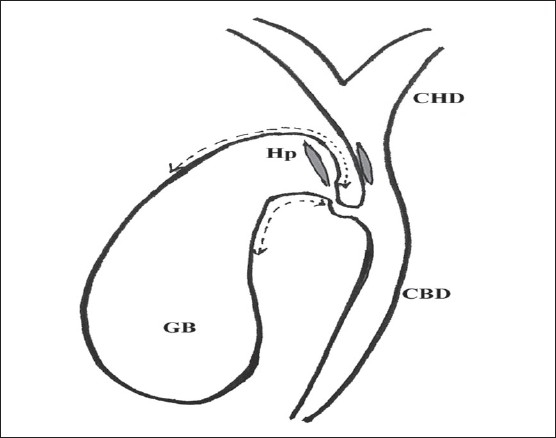
After mobilization (_-----_) of Hartmann's pouch (Hp) from liver bed, the distended common hepatic duct (CHD) and Hartmann's pouch are incised at a length of 15-20 mm. The incised parts are anchored by three transfixing sutures, A-a, B-b and C-c (See figure 2). CHD-Common hepatic duct, Hp-Hartmann pouch, GB-Gallbladder. CBD-Common bile duct.

**Figure 2:**
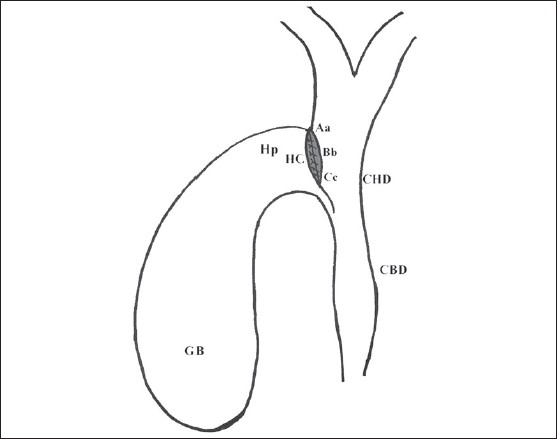
Anastomosis between gallbladder (GB) and the common hepatic duct (CHD) performed by a single layer interrupted 3-0 polyglcolic acid sutures. A-a, B-b and C-c, transfixing sutures in place. HC-Hepaticocholecystostomy.

**Figure 3:**
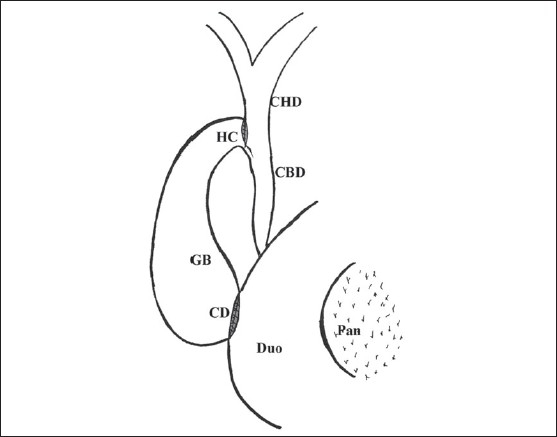
A side-to-side cholecystoduodenostomy (CD) is performed by interrupted 3-0 polyglcolic acid sutures. HC-Hepaticocholecystostomy, GB-Gallbladder, CD-Cholecystoduodenostomy, Duo-Duodenum, CHD-Common hepatic duct, CBD-Common bile duct, Pan-Pancreas, Hp-Hartmann pouch.

Surgical exploration was performed using a right subcostal incision. Once the suitability of the patient for the procedure was ascertained, serosa of the hepatoduodenal ligament was opened and common hepatic and bile ducts exposed, whereupon the distended gallbladder (GB) was seen to lie close to the common hepatic duct (CHD) and the duodenum below. Hartmann pouch was mobilized to facilitate approximation of the CHD and the neck of GB, transfixation sutures were placed between the two structures (Figure 1) A 15-20 mm side to side anastomosis was performed between the CHD and neck of the GB by a single layer of interrupted sutures using 3-0 polyglycolic acid (Figure 2). While performing the anastomosis, due care was taken to avoid injury to the main cystic artery lying in the triangle between the CHD and the cystic duct (cystohepatic triangle). The fundus of the gallbladder was grasped with a noncrushing intestinal clamp and it was freed by careful dissection of the surrounding tissue; the duodenum was also freed from its lateral peritoneal attachments (the Kocher maneuver). A single layer interrupted two to three cm side to side cholecystoduodenostomy was performed in the second part of the duodenum on its anterior aspect well away and below the tumor (Figure 3). After placing a tube drain in Morison pouch, the abdomen was closed in layers. For choledochojejunostomy, the markedly distended GB was removed and a Roux-en-y loop of jejunum was prepared for the classical side to side choledochojejunostomy anastomosis. Bypass was considered a failure if reoperation or other therapeutic intervention was necessitated for managing postoperative complications related to biliary anastomosis or recurrence of jaundice. After leaving the hospital, patients were followed by direct or telephonic contact. The study was approved by the institute ethical committee, and informed consent was obtained from all the patients for participation in the study.

Summary statistics for quantitative data were the mean and standard deviation (SD) presented as “mean (SD)”. Quantitative data between the two treatment groups was compared using the t test for parametric data. All *P* values were two-tailed; *P* values less than .05 were considered statistically significant. Statistical analysis was performed using a statistical software program (SPSS 11.5.0)

## RESULTS

Each group had 10 patients with the mean patient age in group I (HCD group) being 64.1 years (range, 56-72 years) and group II (CDJ group) 61 years (range, 50-72 years). There were 6 females and 4 males in group I, 7 males and 3 females in group II. The chief complaints of patients in either group were itching, jaundice and pain. Preoperatively, USG and CT were useful in detecting advanced lesions in 7 patients of group I and 8 patients of group II. In the other patients, irresectability was ascertained intraoperatively. All patients were subjected to preoperative pathological diagnosis either by endoscopy or FNA. There was no noticeable difference between pre- and postoperative laboratory findings of the two groups (Table 1). A significant decline in serum bilirubin, liver enzymes and jaundice was observed in all the patients postoperatively, with remarkable clinical improvement. On comparison of the operative details, the mean operative time and mean intraoperative blood loss was significantly less in group I (*P*<.05). The length of postoperative enteral feeding and duration of hospital stay was also found significantly less in group I (*P*<.05). Two patients in each group had minor postoperative complications (group I, one wound infection, one left basal atelectasis; group II, one wound infection, one minor anastomotic bile leak) that were managed conservatively. There were no cases of operative mortality or anastomotic leak in either group. The overall survival time was 8.2±3.7 months in group I and 8.1±4.1 months in group II. Two patients in group II and one patient in group I had to be readmitted due to local extension of the tumor causing recurrent jaundice. One patient in group II developed severe gastrointestinal bleeding caused by erosive gastritis and was managed conservatively. Duodenal obstruction occurred in one patient in group I, necessitating an endoscopic stenting.

**Table 1:** Main outcomes of the hepaticocholecystoduodenostomy (HCD) compared with choledochojejunostomy (CDJ).

	Group I (HCD) Mean (SD)	Group II (CDJ)Mean (SD)	*P* value
No. of cases	10	10	
Operative time, min	84.7 (10.3)	133.6 (8.9)	<.0001
Operative blood loss, mL	137.8 (37.2)	201.6 (23.4)	.0002
Onset of enteral feeding, day	3.3 (0.5)	5.0 (0.7)	<.0001
Days of hospitalization	7.5 (0.7)	9.7 (1.2)	<.0001
Serum bilirubin, μmol/L			
Preoperative	165.41 (62.71)	200.77 (69.43)	.005^a^
Postoperative	82.79 (34.42)	105.91 (49.66)	.005^b^
Serum ALP, IU/L			
Preoperative	862.2 (237.7)	998.5 (597.9)	.005^a^
Postoperative	420.8 (136.7)	4.3.6 (280.7)	.005^b^
Serum SGOT, IU/L			
Preoperative	84.2 (23.2)	74.3 (19.6)	.005^a^
Postoperative	54.2 (14.0)	45 (13.8)	.005^b^
Serum SGPT, IU/L			
Preoperative	56.0 (23.6)	52.3 (17.0)	.005^a^
Postoperative	32.0 (7.7)	33.6 (9.0)	.005^b^

Abbreviations used, ALP, alkaline phosphatase; SGOT, serum glutamic. oxaloacetic transaminase; SGPT, serum glutamic pyruvic transaminase

a *P* value in HCD group

b *P* value in CDJ group.

## DISCUSSION

Periampullary adenocarcinoma includes a diverse group of lesions around the ampulla of Vater displaying identical clinical features but associated with different prognoses. Most of such lesions are pancreatic head adenocarcinomas; 5% to 20% of them are resectable for cure at the time of appearance;^4^–^9^ 70% of patients develop obstructive jaundice with pancreatic cancer at some point in their clinical course.^5^–^8^ Biliary obstruction impairs liver function which can lead to hepatic failure, 25% of patients develop agonizing pruritis which cannot be relieved by medical means. Some form of palliation, therefore, becomes necessary to give respite to these patients from their agonizing symptoms and improve the quality of their life.

Patients with periampullary adenocarcinoma are mostly unresectable at the time of diagnosis. Often they need some procedure for relieving the biliary obstruction. Aided by modern radiological modalities, identification of patients with advanced disease forms a subset of patients who may be served better by endoscopic stenting. However, a significant group of patients initially felt to have operable tumors are still found to have unresectable disease upon exploration. Additionally, there is a group of patients in whom either stenting has failed or stents get repeatedly blocked. In such a group surgical biliary bypass becomes mandatory. The aim of any operation undertaken for palliation in patients with unresectable periampullary cancer is to use the simplest procedure with the lowest incidence of immediate complications, such as anastomotic leak and recurrent biliary obstruction requiring reoperation. The accepted decompressive surgical options for treating the malignant distal biliary obstruction are cholecystoenterostomy and choledochoenterostomy.^10^ A review of the literature reveals conflicting opinions on the operation of choice in such patients.^1^,^2^ Advocates of cholecystoenterostomy claim that the operation is simple, quick and capable of relieving jaundice with minimal blood loss 1 and those who side with choledochoenterostomy hold that the gallbladder and cystic duct are not dependable for biliary decompression.^2^,^11^

The use of gallbladder for biliary decompression in periampullary tumors creates an apprehension that the primary tumor may relentlessly creep upwards by direct extension along and around the common bile duct and obstruct it. Addressing this concern, Sarfeh et al concluded that choledochoenterostomy is significantly a more effective method of palliation than one involving the use of the gallbladder; describe it as the procedure of choice.^10^ In a recent study comparing cholecystojejunostomy with choledochojejunostomy in patients with unresectable pancreatic cancer, Urbach et al concluded that there was 4.4 times increase in the risk of requiring a subsequent biliary drainage procedure in the cholecystojejunostomy group.^12^ They also observed that shorter survival in CJ group was possibly related to sepsis due to inadequate biliary drainage noticed in these patients. The patency of the cystic duct, the level of its entry and relationship to the obstructing tumor seem to be the cardinal factors which determine the drainage results when the gallbladder is considered for biliary decompression. Keeping these facts in view, it is recommended that palliative biliary bypass be attempted by choledochojejunostomy rather than by cholecystojejunostomy.

Less operative time, minimal blood loss, early enteral feeding and shorter hospital stay makes the HCD procedure look attractive. While both methods effectively reduce the bilirubin levels, the development of recurrent jaundice seems to be lower after HCD possibly due to lower rate of anastomotic invasion by the tumor cells due to comparatively higher placement of the anastomosis. HCD may also offer a more physiologic operation as against Roux-en-y choledochojejunostomy, since it keeps the duodenum bathed in alkaline secretion, thereby neutralizing gastric acid and maintaining normal feedback mechanism of gastrointestinal secretions. This could result in a lower rate of ulcer formation vis-à-vis Roux-en-y choledochojejunostomy.

One patient in our CDJ study group came down with hemorrhagic gastritis requiring blood transfusion. This again looks to be healthy indicator and compares favorably with other studies where gastrointestinal bleeding following CDJ ranged between 4.7% to 15%.^13^,^14^ It also saves extending the operating field below the mesocolon and importantly in already poorly nourished patients, it does not remove from function a segment of jejunum. A similar type of procedure described by Vankemmel et al has been performed with excellent results in patients with chronic pancreatitis.^15^ In the Vankemmel procedure, after resection of the cystic duct, the neck of the gallbladder is anastomised (end to end) to the common bile duct and later the fundus of the gallbladder is anastomised to the second part of the duodenum. The difference between Vankaemmel's and our procedure is that we mobilize the neck of the gallbladder taking care not to injure the cystic artery and perform a side-to-side anastomosis rather than and end-to-end anastomosis between the common hepatic duct and the neck of the bladder. Thus, the anastomosis is placed higher and the patent cystic duct acts as an additional conduit for the passage of the obstructed bile.

Advanced periampullary adenocarcinoma patients have shorter mean survival time, as such prophylactic gastrojejunostomy seems unnecessary as most of them do not survive long enough to develop late gastric outlet obstruction and where they do, one can resort to endoscopic duodenal stenting. Minimal surgical dissection and manipulation seem to be the important factors involved in early return of bowel sounds.^16^ It has been hypothesized that abdominal surgery initiates a scenario of inflammatory events that results in common clinical phenomenon of post surgical ileus.^17^ Thus, surgical manipulation and the extent of dissection seem to correlate well with the return of bowel sounds. Patients who undergo HCD are subjected to minimal surgical dissection and manipulation and as such experience early return of bowel sounds leading to early return to oral feeding and hence early discharge from the hospital. These visible advantages may lure surgeons to perform HCD in patients lined for a palliative bypass. Recent studies have revealed that laparoscopic bypass, whether cholecystojejunostomy or choledochojejunostomy, is technically feasible, safe and performed within a reasonable period.^18^,^19^ It is conceivable that, in the not too distant future, these laparoscopic techniques can be extended quite efficiently to HCD to make it more effective. Our study suggests that HCD is an effective procedure for palliative decompression in patients with advanced periampullary tumors. However, further research is needed before it can recommended as a standard procedure in such situations.

## References

[1] Sarr MG, Cameron JL (1982). Clinical review: surgical management of unresectable carcinoma of the pancreas. Surg.

[2] Aokia Y, Katusumi M (1984). Palliative surgery for unresectable carcinoma of the head of the pancreas, ampulla and distal end of the common bile duct in Japan. Am J Surg.

[3] Gouma DJ, van Geenen R, van Gulik T, de Wit LT, Obertop H (1999). Surgical palliative treatment in biliapancreatic malignancy. Ann Oncol.

[4] Neuberger TJ, Wade TP, Swope TJ, Virgo KS, Johnson FE (1993). Palliative operation for pancreatic cancer in the hospital of U.S. Department of Veterans Affairs from 1987 to 1991. Am J Surg.

[5] Lillemoe KD, Sauter PK, Pitt HA, Yeo CJ, Cameron JL (1993). Current status of surgical palliation of periampullary carcinoma. Surg Gynecol Obstet.

[6] Singh SM, Longmire WP, Reber HA (1990). Surgical palliation for pancreatic cancer: the UCLA experience. Ann Surg.

[7] Lillemoe KD, Pitt HA (1996). Palliation: Surgical and otherwise. Cancer.

[8] Doberneck RC, Berndt GA (1987). Delayed gastric emptying after palliative gastrojejunostomy for carcinoma of the pancreas. Arch Surg.

[9] Huguier M, Baumel H, Manderschied JC, Houry S, Fabre JM (1993). Surgical palliation for unresected cancer of the exocrine pancreas. Eur J Surg Oncol.

[10] Sarfeh J, Rypins EB, Jakowatz JG, Juler GL (1998). A prospective, randomized clinical investigation of cholecystoenterostomy and choledochoenterostomy. Am J Surg.

[11] Brooks DC, Osteen RT, Gray EB, Steele GD, Wilson RE (1981). Evaluation of palliative procedures for pancreatic cancer. Am J Surg.

[12] Urbach DR, Bell CM, Swanstrom LL, Hansen PD (2003). Cohort Study of Surgical bypass to thegallbladder or bile duct for the palliation of jaundice due to pancreatic cancer. Ann Surg.

[13] Deziel DJ, Wilhelmi B, Staren ED, Doolas A (1996). Surgical palliation for ductal adenocarcinoma of the pancreas. Am Surg.

[14] Sarr MG, Gladen HE, Beart RW, Van Heerden JA (1981). Role of gastroenterostomy in patients with unresectable carcinoma of the pancreas. Surg Gynecol Obstet.

[15] Vankemmel M, Martin F, Baspeyre H, Dupuys F (1990). Bilio-digestive bypass using gallbladder in chronic pancreatitis. 85 cases of cholecystoplasty. Chirurgie.

[16] Garcia-Caballero M, Vara-Thornbeck C (1993). The evolution of postoperative ileus after laparoscopic cholecystectomy. Surg Endosc.

[17] Kalff JC, Schrant WH, Simmous RL, Bauer AJ (1998). Surgical manipulation of the gut elicits an intestinal muscularis inflammatory response resulting in post surgical ileus. Ann Surg.

[18] Brune IB, Feussner H, Neuhaus H, Classen M, Siewert JR (1997). Laparoscopic gastrojejunostomy and endoscopic biliary stent placement for palliation of incurable gastric outlet obstruction with cholestasis. Surg Endosc.

[19] Bergamaschi R, Marvik R, Thoresen JF, Ystgaard B, Johnsen G, Myrvold HE (1998). Open versus laparoscopic gastrojejunostomy for palliation in advanced pancreatic cancer. Surg Laparosc Endosc.

